# Added value of pre-procedural magnetic resonance angiography in transarterial embolization for refractory musculoskeletal pain

**DOI:** 10.3389/fmed.2024.1471504

**Published:** 2024-11-06

**Authors:** Chiao-Yun Pan, Keng-Wei Liang, Ting-Rong Chen, Chien-Kuo Wang, Wen-Ying Liao, Ying-Hung Lu, Yi-Cheng Hsiung, Yi-Sheng Liu, Bow Wang

**Affiliations:** ^1^Department of Medical Imaging, National Cheng Kung University Hospital, College of Medicine, National Cheng Kung University, Tainan, Taiwan; ^2^Department of Medical Imaging, Chung Shan Medical University Hospital, Taichung, Taiwan; ^3^Interventional Medicine Center, National Cheng Kung University Hospital, College of Medicine, National Cheng Kung University, Tainan, Taiwan; ^4^Department of Biotechnology and Bioindustry Sciences, National Cheng Kung University, Tainan, Taiwan

**Keywords:** joints, magnetic resonance angiography, angiogenesis, musculoskeletal pain, embolization, therapeutic

## Abstract

**Background:**

Transarterial microembolization (TAME) is a minimally invasive treatment for chronic musculoskeletal disorders. Identifying angiogenesis and the supplying vessels of the target joint is important but challenging. Although magnetic resonance imaging (MRI) is commonly used to diagnose musculoskeletal diseases, it typically excludes vascular imaging. Dynamic contrast-enhanced magnetic resonance angiography (DCE-MRA) has the ability to visualize lesion angiogenesis, identify supplying vessels, and evaluate the vasculature anatomy. We propose that incorporating DCE-MRA into pre-procedural assessments can help identify the culprit vessels, arterial anatomy, and variant assessment of the target joint before TAME.

**Materials and methods:**

We investigated six cases, each presenting pain in different body parts: shoulder adhesive capsulitis, trapezius myalgia, combined tennis and golf elbow, knee osteoarthritis, refractory knee pain after osteotomy, and plantar fasciitis. All patients underwent MRI with DCE-MRA before undergoing TAME. DCE-MRA was performed using either 1.5 T or 3 T MRI scanners, employing 3D-TRICKS or 4D-TRAK XD techniques. The numerical rating scale for pain was evaluated at one, three, and six months after the procedure, and any adverse events were recorded over the entire six-month follow-up period.

**Results:**

Pre-procedural DCE-MRA helped to visualize angiogenesis at the lesion site in all patients and identify the supplying vessels, arterial vasculature anatomy, and branching variants. These findings corroborated the subsequent digital subtraction angiography (DSA) findings obtained during TAME. All patients experienced pain reduction and functional improvement after TAME without any complications. The average pain score reduced significantly after TAME treatment (*p* < 0.05). Two patients underwent a second MRI and DCE-MRA at the six-month follow-up and showed a significant reduction in angiogenesis.

**Conclusion:**

DCE-MRA offers a valuable pre-procedural assessment tool for TAME procedures by facilitating the visualization of angiogenesis at the lesion site, supplying vessels, and arterial anatomic variants, including the variable orifice of the supplying branches. This information can potentially improve patient selection and pre-procedural planning, leading to better outcomes and reduced risk of complications.

## 1 Introduction

Transarterial microembolization (TAME), first introduced by Okuno et al. (1), is increasingly being recognized as a minimally invasive treatment for chronic musculoskeletal disorders. Recently, several narrative and systematic reviews have concluded that TAME is a safe and effective treatment option for patients with chronic joint pain (2–5).

Although TAME is considered safe, several rare complications have been reported. Lin et al. (6) broadly categorized these complications into intra-interventional challenges, such as microarterial perforation, dissection, or other iatrogenic vascular injuries, and post-interventional complications, such as limb ischemia.

Careful patient selection and meticulous pre-procedural planning are essential to minimize complications in vascular interventions. Pre-procedural mapping of the vascular anatomy is crucial for accurately identifying the target vessels and avoiding inadvertent embolization of non-target vessels. However, no established standard protocol exists currently for the pre-procedural evaluation of TAME.

Embolization of abnormal angiogenesis at the lesion site is the central strategy in TAME therapy (7). The presence of these neo-vessels in the pre-procedural images supports the indications for TAME (8). Conversely, the presence of advanced atherosclerosis may render TAME an unsuitable treatment option (1). According to the randomized controlled trial by Sasanuma et al. (9), dynamic contrast-enhanced magnetic resonance angiography (DCE-MRA) enables visualization of the abnormal neovascularization in cases of symptomatic rotator cuff tears and frozen shoulders. DCE-MRA also demonstrates high sensitivity (95–100%) and specificity (98–100%) for detecting stenosis and occlusions in patients with peripheral arterial occlusive disease and shows excellent correlation with digital subtraction angiography (DSA) (10). Additionally, DCE-MRA has been proven effective in diagnosing various vascular anomalies in the upper and lower limbs (11, 12).

Accurately identifying the culprit arteries of the target joint is essential to ensure effective TAME. DCE-MRA can accurately identify feeding arteries when assessing arteriovenous malformations or arteriovenous fistulas in the extremities (13). However, the variability in vascular branching patterns can lead to prolonged procedure times or missed identification of critical pathogenic vessels, thereby reducing treatment effectiveness. For example, Okuno et al. (14) found that the suprascapular artery (SSA), an important vessel implicated in shoulder pain, could exhibit five distinct branching variants. DCE-MRA has also been validated as a preoperative tool to evaluate vascular variations during fibular graft transplantation (13).

Based on these considerations, we incorporated DCE-MRA into our pre-TAME evaluation protocol. We propose that it can provide a more comprehensive pre-procedural evaluation, including assessing the angiogenesis of the target lesion joint, identifying supplying vessels, evaluating arterial branching patterns, and detecting arterial abnormalities, such as stenosis, tortuosity, and atherosclerosis, before performing TAME.

## 2 Materials and equipment

### 2.1 MRI scan

Five patients were scanned using a 3 T MR scanner (Ingenia, Philips, Best, The Netherlands), and one patient was scanned using a 1.5 T MRI scanner (Signa Artist, GE Healthcare, Illinois, United States) with compatible body coils.

### 2.2 Contrast medium

An automated power injector (OptiStar^®^ Elite MR injector, Cincinnati, United States) was used. Gadobutrol (gadolinium-DO3A-butriol; Gadovist^®^ 1.0, Schering, Berlin, Germany) was injected initially at a flow rate of 2 mL/s, followed by a saline flush of 20 mL at a flow rate of 2.0 mL/s. The total dose of gadobutrol administered was 0.15 mmol/kg.

## 3 Method

### 3.1 DCE-MRA

DCE-MRA was performed using a sequential T1-weighted 4D-TRAK XD MRA sequence on a 3 T MR scanner (Ingenia, Philips, Best, The Netherlands) and 3D-TRICKS MRA on a 1.5 T MR scanner (GE Healthcare Signa Artist, Illinois, United States).

4D-TRAK XD was performed using the CENTRA keyhole method, with 25% of k-space data sampled in the coronal plane. The central space was randomly filled during the entire passage of the contrast bolus over time, and the periphery of the k-space was collected at 33% of the reference dataset for each dynamic scan. Sensitivity encoding was used with acceleration factors of 3.2 and 1.3 in the phase-encoding and slice-encoding directions, respectively, yielding a total acceleration factor of 4.16. The acquisition parameters of the 4D-TRAK XD technique were as follows: 3D-T1 weighted-gradient echo sequence with repetition time/echo time, 4 ms/2 ms; flip angle, 25°; slice thickness, 3.0 mm; and acquisition time, 120 s (reference scanning time, 15 s; 4 s per phase for 25 phases in total). The 4D-TRAK XD sequence was initiated immediately after starting the injection. The temporal resolution was 4 s per phase, with a total scan time of 180 s.

The 3D-TRICKS imaging parameters were as follows: repetition time/echo time, 4 ms/2 ms; flip angle, 30°; slice thickness, 3.0 mm interpolated to 1.5 mm; and bandwidth, 100 MHz. The temporal resolution was 4 s for each phase, and 15 phases were acquired in total within 2 min.

### 3.2 TAME procedure

TAME was performed by a board-certified interventional radiologist (BW) between May 2023 and May 2024 for the participants. The patient’s pain site was pre-procedurally marked on the skin using a metallic marker. DSA was performed using a biplane fluoroscopy platform (Artis zee; Siemens Healthineers, Erlangen, Germany). Super-selective embolization was performed by manually injecting 3 mL of contrast medium at 1 mL/s via an angiographic catheter (J-curve; 4 Fr × 0.038″ × 65 cm, Terumo, Vietnam) and a microcatheter (Veloute; 1.7 Fr, Asahi Intecc, Thailand). Imipenem/cilastatin sodium was used as the embolic material (Amgen, Culin, Taiwan). A suspension of 0.5 g of imipenem/cilastatin sodium in 10 mL of the iodinated contrast agent was injected. The embolization endpoint was the suppression of or reduction in the filling of the abnormal vessels visible on MRA.

### 3.3 Follow-up

At baseline and the one-, three-and six-month follow-up after TAME, the pain levels were assessed using the numerical rating scale (NRS). The patients rated their pain on the NRS from 0 (no pain) to 10 (worst possible pain) (15). The NRS score was recorded before and after the treatment. The mean and standard deviation of the pre-and post-treatment NRS scores were calculated, and a paired t-test was performed using IBM SPSS Advanced Statistics, version 13.0, to determine the statistical significance of the difference in pain levels. *p*-values <0.05 were considered statistically significant.

Adverse events were documented from the date of the procedure to the six-month follow-up. The recorded items included common TAME complications as mentioned by Lin et al. (6) intra-interventional challenges included microartery perforation, microcatheter tip fracture, and iatrogenic large-artery injuries. Post-interventional complications comprised subcutaneous hemorrhage, changes in skin color, severe evoked pain, transient radial artery spasm, fever, hematoma at the puncture site, skin ulceration, pain at the puncture site, transient increase in pain, transient skin necrosis, transient paresthesia, transient osteomedullary edema, and transient hearing difficulty.

## 4 Case descriptions

### 4.1 Adhesive capsulitis (case 1)

A 57-year-old male with left shoulder pain, clinically diagnosed as adhesive capsulitis, had previously undergone medical treatment and rehabilitation without success. Physical examination revealed that the pain worsened with forward extension, and the NRS score was 6.

**Figure 1:**
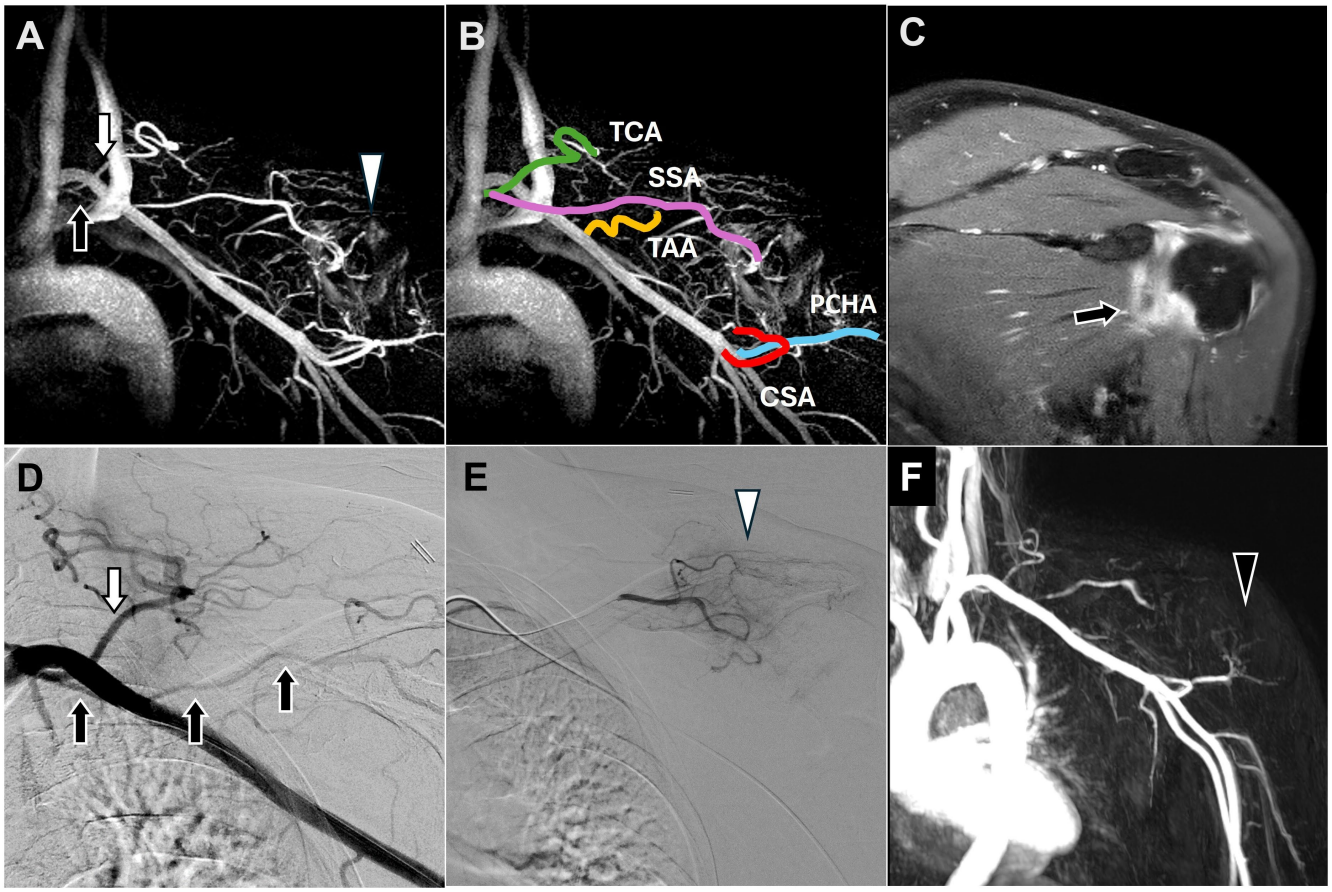
Dynamic contrast-enhanced magnetic resonance angiography and digital subtraction angiography images of adhesive capsulitis of the left shoulder joint. **(A)** Post-contrast T1 fat-saturated images revealed capsular thickening and enhancement of the rotator interval and axillary pouch (black arrow), consistent with the clinical diagnosis of adhesive capsulitis. **(B)** All vascular branches of the shoulder joint involved in TAME were depicted on DCE-MRA. **(C)** The SSA (black arrow), arising from the thyrocervical trunk (white arrow), was identified along with angiogenesis (white arrowhead) surrounding the shoulder joint on DCE-MRA, corroborating the DSA findings in **(D,E)**. **(D)** The DSA showed the SSA (black arrow) arising from the thyrocervical trunk (white arrow). **(E)** The selective DSA via the SSA showed angiogenesis surrounding the shoulder joint (white arrowhead). **(F)** Angiogenesis in the left shoulder was significantly reduced (black arrowhead) at the 6-month follow-up DCE-MRA after TAME. (DCE-MRA, dynamic contrast-enhanced magnetic resonance angiography; DSA, digital subtraction angiography; TAME, transarterial microembolization; TCA, transverse cervical artery; SSA, suprascapular artery; TAA, thoracoacromial artery; CSA, circumflex scapular artery; PCHA, posterior circumflex humeral artery).

MRI of the left shoulder demonstrated hyperintensity and thickening of the coracohumeral and inferior glenohumeral ligaments along with enhancement of the rotator interval and axillary capsule, consistent with adhesive capsulitis of the left shoulder (Figure 1A).

DCE-MRA identified angiogenesis in the left shoulder, the blood vessels supplying blood to the shoulder (Figure 1B), and the SSA branching from the thyrocervical trunk (Figure 1C), consistent with the Type B classification described by Okuno et al. (14).

TAME was performed after 2 months. DSA also revealed the SSA branching from the thyrocervical trunk (Figure 1D) and angiogenesis in the left shoulder (Figure 1E). TAME was performed for the left SSA, left thoracoacromial artery, left circumflex scapular artery, left anterior circumflex humeral artery, and left posterior circumflex humeral artery (16).

The postoperative NRS scores indicated a substantial improvement from 6 to 1 at the 6-month follow-up, with no complications. Repeat left shoulder MRI performed 3 months after TAME demonstrated a significant decrease in angiogenesis (Figure 1F).

### 4.2 Trapezius myalgia (case 2)

A 65-year-old female having a history of trapezius myalgia presented symptoms of right upper back and shoulder pain with an NRS score of 7.

**Figure 2:**
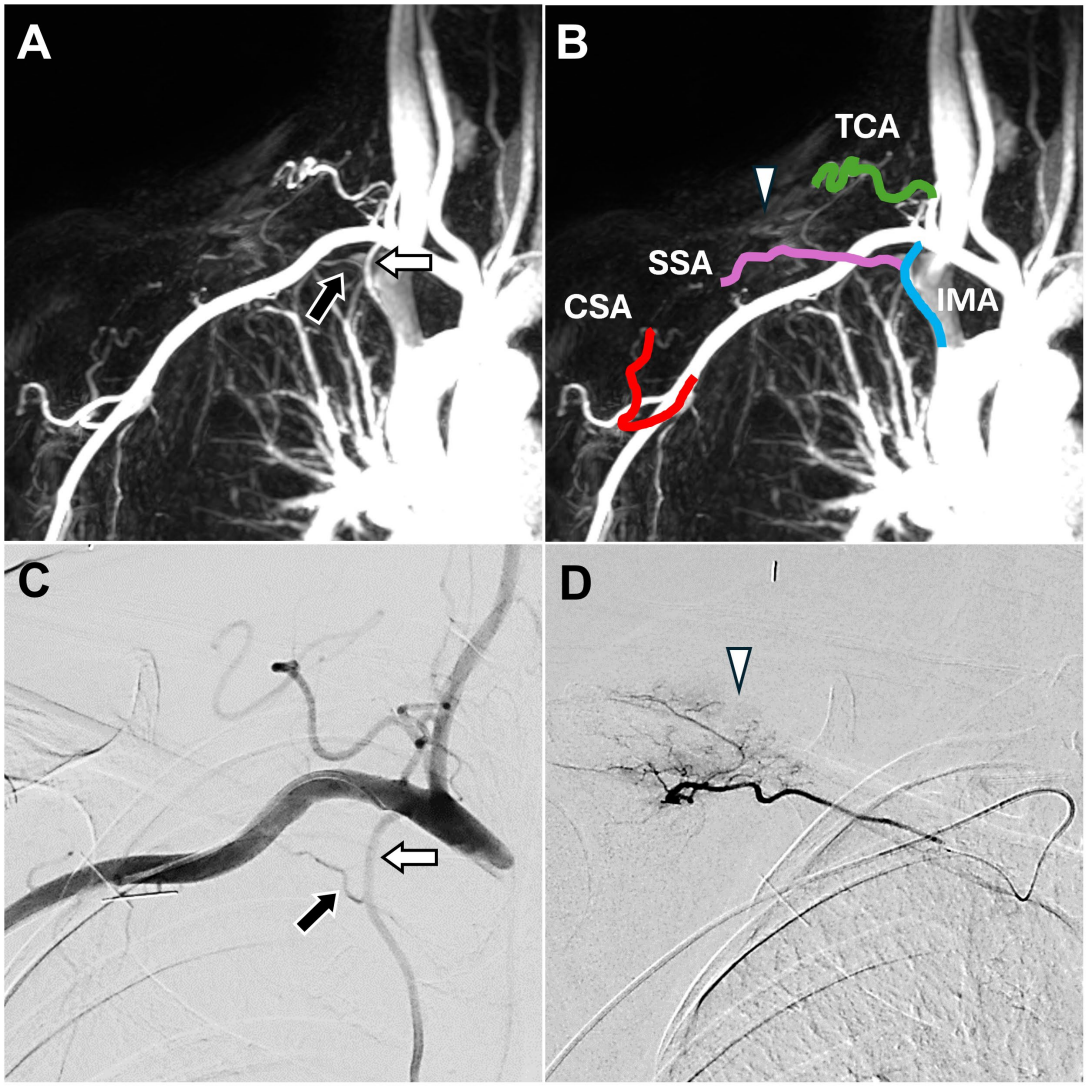
Dynamic contrast-enhanced magnetic resonance angiography and digital subtraction angiography images of trapezius myalgia in the right upper back. **(A)** The SSA (black arrow), arising from the IMA (white arrow), was identified, corroborating the DSA findings in **(C)**. **(B)** DCE-MRA also depicted all TAME-engaged vascular branches, including the right IMA, right SSA, right TCA, and right CSA. Angiogenesis (white arrowhead) was observed in the upper back and shoulder, consistent with the DSA findings in **(D)**. **(C)** The DSA via the subclavian artery showed the SSA (black arrow) arising from the IMA (white arrow). **(D)** The DSA via the SSA showed angiogenesis (white arrowhead) in the upper back and shoulder. (DCE-MRA, dynamic contrast-enhanced magnetic resonance angiography; DSA, digital subtraction angiography; TAME, transarterial microembolization; IMA, internal mammary artery; SSA, suprascapular artery; TCA, transverse cervical artery; CSA, circumflex scapular artery).

DCE-MRA images revealed that the SSA branched from the internal mammary artery (Figure 2A), which is consistent with the Type F classification described by Okuno et al. (14). Angiogenesis was also observed in the right upper back and shoulder (Figure 2B).

DSA confirmed that the SSA branched from the internal mammary artery (Figure 2C) and showed angiogenesis (Figure 2D) at the pain site in the right shoulder. TAME was performed for the right SSA, right transverse cervical artery, and right circumflex scapular artery (17). During the embolization process, the evoked pain corresponded to the pain site.

No complications were observed post-TAME. During the six-month follow-up, the patient’s pain score decreased to 3 on the NRS.

### 4.3 Combined tennis and golf elbow (case 3)

A 60-year-old male presented with left elbow pain persistent for 2 years, with an NRS score of 9. Although the patient tried treatment with painkillers, rehabilitation, acupuncture, local administration of steroids, and platelet-rich plasma injections, the symptoms persisted.

**Figure 3:**
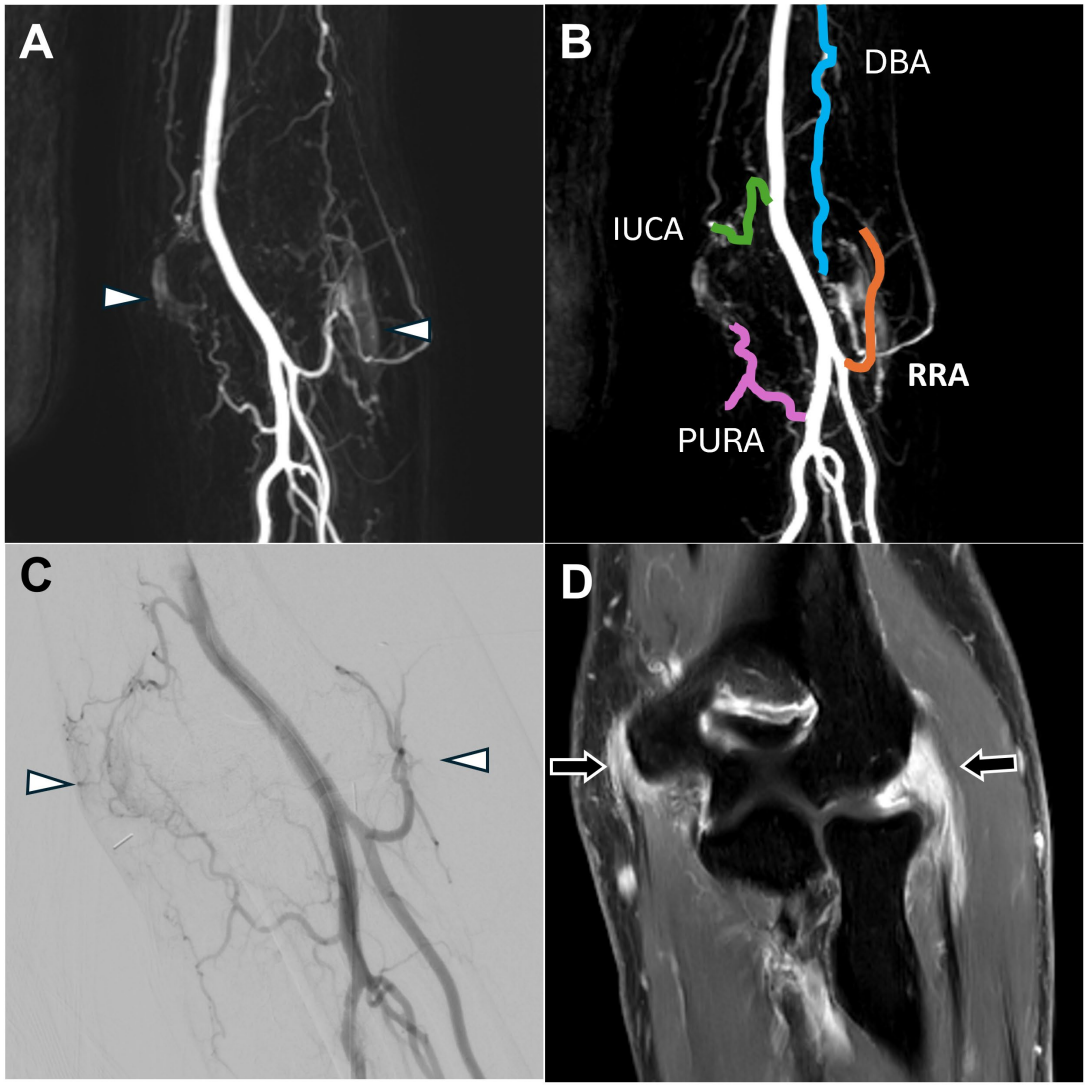
Dynamic contrast-enhanced magnetic resonance angiography and digital subtraction angiography images of both lateral and medial epicondylitis in the right elbow joint. **(A)** Post-contrast T1 fat-saturated images revealed enhancement of the common flexor tendon on the medial side of the elbow and the common extensor tendon on the lateral side (black arrows), indicating tendinopathy. **(B)** Angiogenesis was evident in both the lateral and medial aspects of the elbow joint (white arrowhead), consistent with the DSA findings in **(D)**. **(C)** DCE-MRA clearly depicted the vascular branches supplying the elbow, including the IUCA, RRA, PURA, and DBA. **(D)** The DSA via the distal brachial artery showed angiogenesis in both the lateral and medial aspects of the elbow joint (white arrowhead). (DCE-MRA, dynamic contrast-enhanced magnetic resonance angiography; DSA, digital subtraction angiography; IUCA, inferior ulnar collateral artery; RRA, radial recurrent artery; PURA, posterior ulnar recurrent artery; DBA, deep brachial artery).

MRI revealed fiber disruption with increased signal intensity and thickening of the common extensor tendon at the humeral insertion site and radial collateral ligament, indicating lateral epicondylitis. Additionally, fiber disruption with increased signal intensity and thickening of the common flexor tendon at the humeral insertion site were observed, indicating medial epicondylitis (Figure 3A).

DCE-MRA demonstrated angiogenesis in both the common extensor and common flexor tendon insertion sites of the elbow joint (Figure 3B) as well as the vascular supply to both sides of the elbow (Figure 3C).

DSA confirmed the angiogenesis and vascular branch distribution observed on MRA (Figure 3D). TAME was performed for the left inferior ulnar collateral artery, left posterior ulnar recurrent artery, left radial recurrent artery, and left deep brachial artery (18, 19).

Postoperative follow-up revealed no complications, and the NRS score decreased to 2 at the six-month follow-up.

### 4.4 Knee osteoarthritis (case 4)

An 83-year-old female with right knee osteoarthritis presented with persistent knee pain for 5 years, with an NRS score of 7. The patient’s right knee pain worsened and was refractory to oral analgesics. Physical examination revealed a restricted range of motion in the right knee. The patient opted for non-surgical intervention and pursued further pain management.

**Figure 4:**
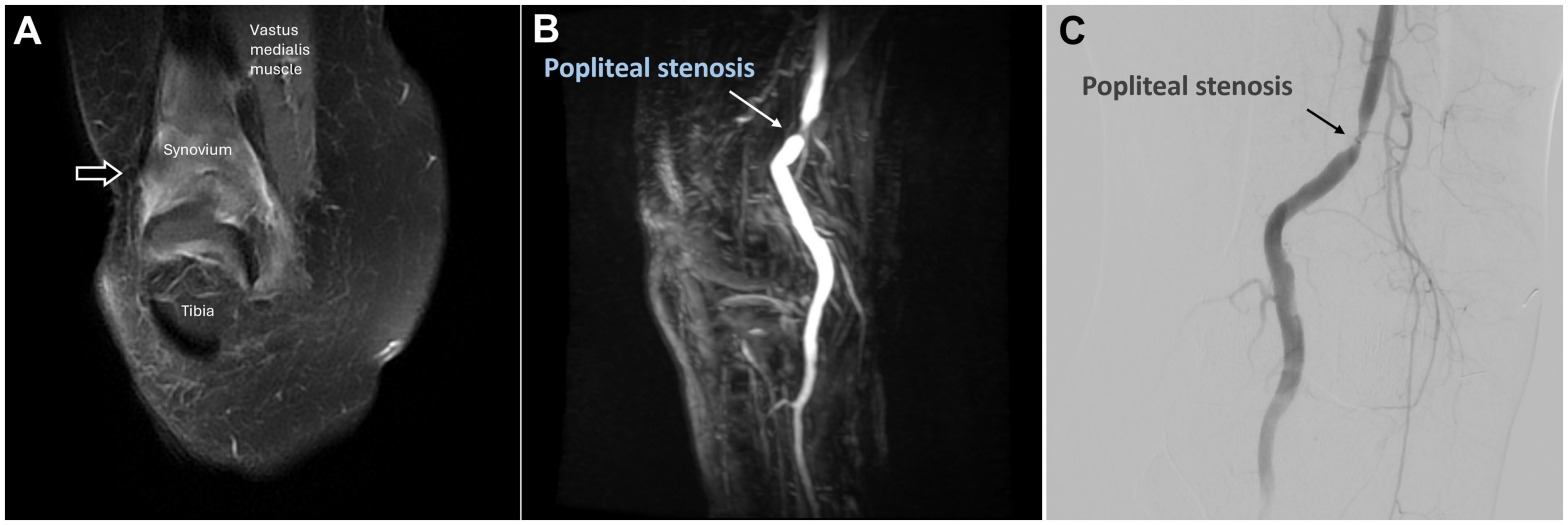
Dynamic contrast-enhanced magnetic resonance angiography and digital subtraction angiography of osteoarthritis in the right knee joint. **(A)** Post-contrast coronal T1-weighted images demonstrated enhancement of the right knee synovium (black arrow). **(B)** DCE-MRA revealed focal stenosis of the popliteal artery (white arrow), which was further corroborated by the DSA findings in **(C)**. **(C)** The DSA via the proximal popliteal artery showed focal stenosis (black arrow). (DCE-MRA, dynamic contrast-enhanced magnetic resonance angiography; DSA, digital subtraction angiography).

MRI showed medial joint space narrowing with cartilage loss, spur formation, mild subchondral bone edema, and enhancement of the synovium, indicative of osteoarthritis in the right knee (Figure 4A).

DCE-MRA with the 3D-TRICK technique revealed abnormal neo-vessels. Severe stenosis of the popliteal artery was also observed (Figure 4B).

According to the DCE-MRA, the 4-French guiding catheter was engaged proximal to the popliteal artery stenosis to avoid vascular injury, and DSA confirmed the popliteal artery stenosis (Figure 4C). Owing to the stenosis severity, super-selective embolization of the genicular arteries was achieved using a 1.7-French Veloute microcatheter (Asahi) to navigate the stenosis within the vessel. The procedure was concluded smoothly without any complications such as dissection. After 6 months, the patient’s NRS score had decreased to 4.

### 4.5 Refractory knee pain after osteotomy (case 5)

A 59-year-old male with a history of right high tibial osteotomy presented with bilateral knee and calf pain after years of squatting, with an NRS score of 8. The patient had undergone rehabilitation for approximately 5 months without any pain relief.

**Figure 5:**
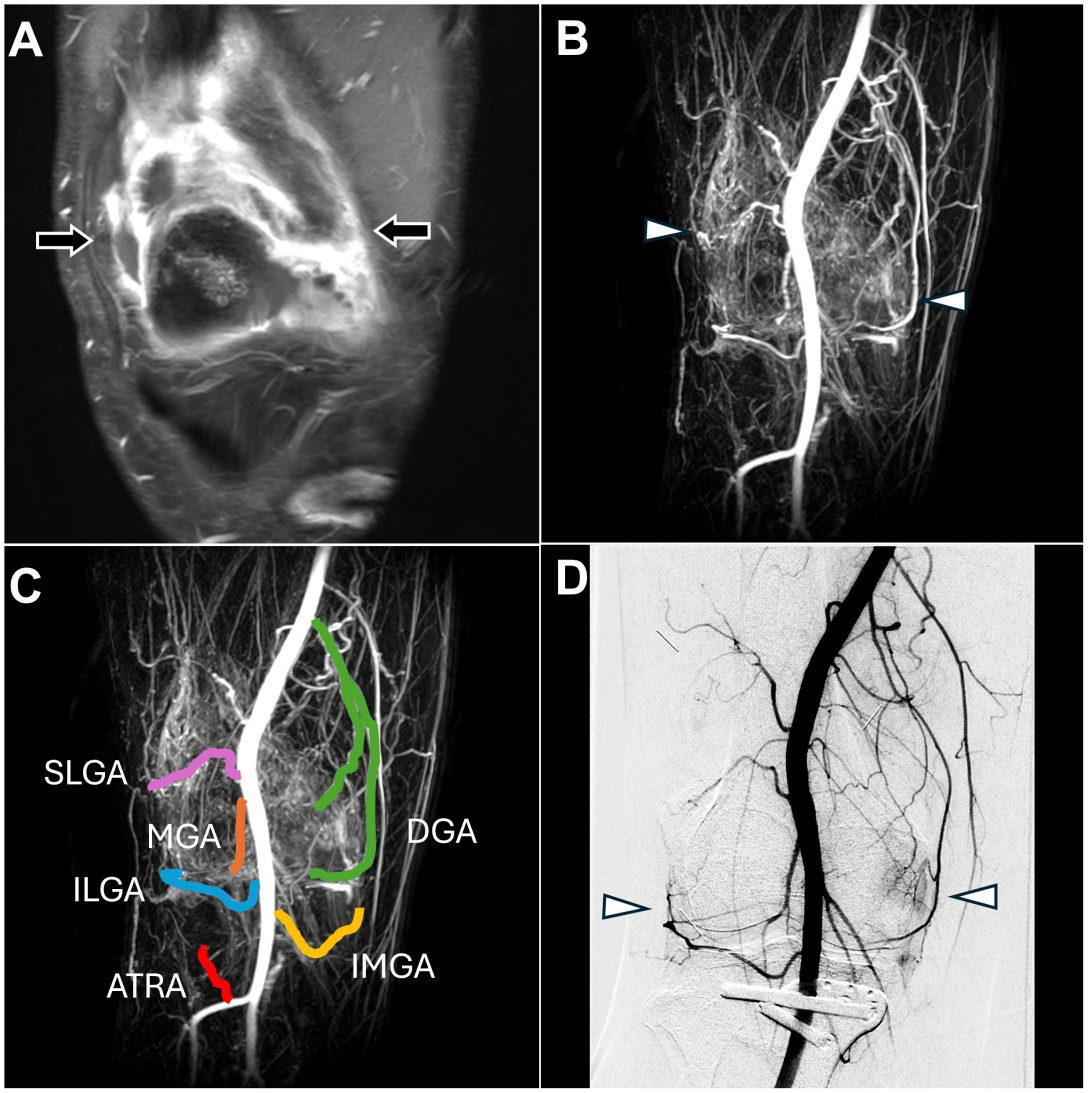
Dynamic contrast-enhanced magnetic resonance angiography and digital subtraction angiography images of refractory right knee pain after osteotomy. **(A)** Post-contrast fat-saturated T1-weighted images demonstrated synovitis with enhancement of the right knee synovium (black arrow). **(B)** DCE-MRA showed angiogenesis in the right knee synovium (white arrowhead), consistent with the DSA findings in **(D)** and enhanced MRI findings in **(A)**. **(C)** DCE-MRA depicted all TAME-engaged vascular branches, including the SLGA, ILGA, MGA, ATRA, DGA, and IMGA. **(D)** The DSA via the popliteal artery showed angiogenesis surrounding the knee joint (white arrowhead). (DCE-MRA, dynamic contrast-enhanced magnetic resonance angiography; DSA, digital subtraction angiography; TAME, transarterial microembolization; SLGA, superior lateral genicular artery; ILGA, inferior lateral genicular artery; MGA, median genicular artery; ATRA, anterior tibial recurrent artery; DGA, descending genicular artery; IMGA, inferior medial genicular artery).

MRI revealed synovial thickening with enhancement and effusion, indicating synovitis of the right knee joint (Figure 5A).

DCE-MRA demonstrated angiogenesis in the right knee synovial membrane (Figure 5B) and vascular supply to the knee (Figure 5C).

DSA confirmed the angiogenesis and vascular supply observed on MRA (Figure 5D). TAME was performed for the right superior lateral genicular artery, right inferior lateral genicular artery, right median genicular artery, right anterior tibial recurrent artery, inferior medial genicular artery, and right descending genicular artery (20). The procedure was completed without any complications, and the NRS score decreased to 6 during the follow-up.

### 4.6 Plantar fasciitis (case 6)

A 62-year-old male had a painful disability in the left heel for over 2 years. Despite conservative treatment, including painkillers, multiple local injections, and shock wave therapy, the patient experienced no significant pain relief; the NRS score was 9.

**Figure 6:**
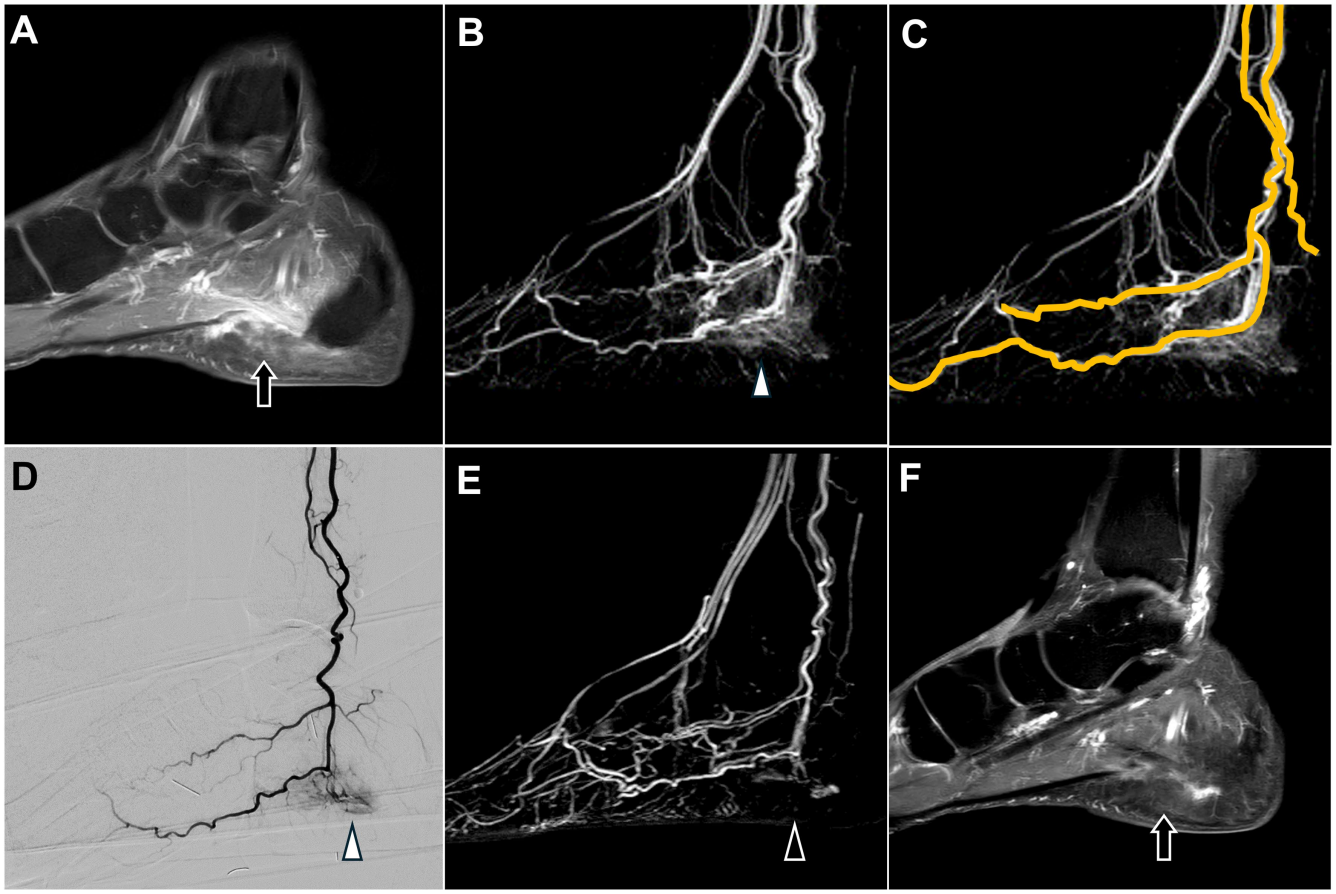
Dynamic contrast-enhanced magnetic resonance angiography and digital subtraction angiography of plantar fasciitis in the left foot. **(A)** Post-contrast T1 fat-saturated images revealed swelling and enhancement of the plantar fascia, consistent with the clinical diagnosis of plantar fasciitis (black arrow). **(B)** DCE-MRA showed angiogenesis in the calcaneal plantar region (white arrowhead), consistent with the DSA findings in **(D)**. **(C)** DCE-MRA depicted the course of the left posterior tibial artery. **(D)** The selective DSA via the posterior tibial artery shows angiogenesis surrounding the calcaneal plantar region (white arrowhead). **(E,F)** Follow-up MRA six months after TAME demonstrates a significant reduction in angiogenesis (black arrowhead) and decreased swelling and enhancement of the plantar fascia (black arrow). (DCE-MRA, dynamic contrast-enhanced magnetic resonance angiography; DSA, digital subtraction angiography; TAME, transarterial microembolization; MRA, magnetic resonance angiography).

MRI revealed swelling and enhancement of the plantar fascia (Figure 6A) as well as edema of the adjacent fat pad and underlying soft tissues, indicating plantar fasciitis.

DCE-MRA revealed angiogenesis in the plantar region (Figure 6B), with blood supply primarily from the left posterior tibial artery (Figure 6C).

DSA confirmed angiogenesis in the plantar region and its supply by the left posterior tibial artery, as observed on MRA (Figure 6D). TAME was performed for the left posterior tibial artery (21).

No postoperative complications were reported, and the patient’s NRS score decreased to 2. A follow-up MRI with DCE-MRA performed after 6 months demonstrated a marked reduction in the angiogenesis in the left plantar region (Figure 6E). Additionally, MRI revealed decreased swelling and enhancement of the plantar fascia (Figure 6F), suggesting alleviation of the inflammation.

## 5 Results

This study included six patients, comprising four males and two females. The average age of the patients was 64.33 ± 9.54 years. The mean pre-treatment NRS score was 7.67 ± 1.21, indicating a high level of pain across the patient cohort. Post-treatment, the mean NRS score dropped to 3.00 ± 1.79, reflecting a substantial reduction in the reported pain levels. The paired *t*-test revealed that the decrease in pain was statistically significant at a *p*-value of 0.003.

By the end of the six-month follow-up period, no patient experienced any potential complications associated with TAME, as documented in the study.

## 6 Discussion

Non-contrast MRI, with its ability to provide detailed images of soft tissues, joints, and bones, is a valuable tool for the clinical diagnosis of musculoskeletal disorders. Studies have demonstrated the utility of MRI in pre-TAME assessments, enabling the evaluation of full-thickness cartilage defects, effusion synovitis, bone marrow lesions with subchondral insufficiency fractures of the knee, osteophytes, and cartilage surface area scores, which can serve as poor prognostic factors for TAME outcomes (22–24). However, vascular imaging is not routinely incorporated into the pre-TAME MRI protocols. This study aimed to illustrate the potential value of incorporating DCE-MRA into the pre-TAME evaluation.

Neovascularity is associated with pain manifested by osteoarthritis and synovitis (25, 26). Lesions with abnormal neovascularization and a high degree of angiographic enhancement may indicate better treatment outcomes, thereby reinforcing the indications of TAME (7, 8). In the six cases presented in this study, DCE-MRA successfully visualized angiogenesis at the lesion sites in all patients, which corresponded with the patients’ pain locations and matched the blushing locations observed on DSA. All patients experienced pain relief after TAME. Consequently, the visualization of angiogenesis using DCE-MRA may indicate that TAME could be a viable therapeutic approach for alleviating pain.

Multiple variations in vascular openings can make the TAME procedure more challenging and time-consuming and consequently increase radiation exposure for both the patient and operator as well as the likelihood of complications. The limitations of DSA without a pre-procedural vascular mapping technique include difficulty in identifying diminutive, tortuous, or overlapping vessels (27). Considering the numerous variations in the vascular branches of the shoulders and knees (14, 28), establishing a pre-procedural vascular map can facilitate smooth execution of TAME. With advancements in imaging technology, DCE-MRA offers good spatial resolution, allowing clearer imaging of the small vessels in the limbs (11, 12). In our six cases, we demonstrated that DCE-MRA could identify the vessels requiring TAME and recognize different variations in vascular branching. During embolization, the evoked pain confirmed that the embolized vessels were the target arteries.

All patients in this study underwent successful TAME without complications (6). In case 4, pre-procedural DCE-MRA identified vascular stenosis, allowing careful use of a guiding catheter and microcatheter to navigate the stenosis for embolization. This illustrates how DCE-MRA can assist in pre-procedural treatment planning and avoiding complications. Minor complications, such as transient increases in pain, transient skin necrosis, and transient paresthesia, were not observed. We hypothesized that this could be attributed to the pre-procedural DCE-MRA, which enabled us to perform super-selective embolization, the use of imipenem/cilastatin as a temporary embolization agent, and the administration of a relatively low therapeutic dose.

Additionally, DCE-MRA can be used as a non-invasive follow-up tool. In cases 1 and 6, a marked reduction in angiogenesis and enhancement of the capsule and fascia were observed at the six-month follow-up. These findings are consistent with the significant improvement in the clinical pain symptoms reported by the patients.

Compared with computed tomography angiography, DCE-MRA offers the advantages of being radiation-free and avoiding contrast-induced nephropathy. In comparison to time-of-flight MRA, a traditional MRA technique for vascular imaging without a contrast medium, DCE-MRA exhibits superior temporal resolution, is less dependent on vessel orientation, requires shorter scanning times, and produces fewer artifacts (29, 30). Although positron emission tomography-computed tomography can confirm inflammation at the pain site and shows a significant reduction in fluorodeoxyglucose uptake following TAME, the radiation exposure of the patient should be considered (31). Additionally, positron emission tomography-computed tomography cannot provide vascular mapping images, which are crucial for pre-procedural planning and treatment guidance.

DCE-MRA has several potential drawbacks, including increased healthcare costs and the limitation in advanced imaging equipment. Additionally, the use of gadolinium-based contrast agents (GBCA) may raise concerns regarding allergic reactions or nephrogenic systemic fibrosis. Notably, the contrast agent employed in this study was a Group II non-ionic linear GBCA. A previous meta-analysis revealed that non-ionic linear GBCAs exhibited the lowest overall rate of immediate adverse reactions at 1.5 per 10,000 administrations (32). Another study indicated that the risk of nephrogenic systemic fibrosis associated with Group II GBCA administration in patients with Stage 4 or 5 chronic kidney disease is less than 0.07% (33). Despite the extremely low incidence of adverse effects, specific patient groups, such as pregnant women, breastfeeding mothers, or children, may require additional consideration when receiving contrast agents. In such cases, the lowest possible dose should be used.

This is the first study introducing the use of DCE-MRA as a part of the pre-procedural assessment for TAME. The primary purpose of this study was to present six case reports of patients who underwent pre-TAME DCE-MRA examinations and subsequently received successful TAME treatment. We collected data of cases of five different anatomical sites to demonstrate the applicability of DCE-MRA at various locations. However, this study has some limitations owing to its small sample size and the potential for selection bias inherent in the case series. To evaluate the safety and effectiveness of DCE-MRA as a pre-TAME screening tool more comprehensively, future research with a larger patient cohort is required. Furthermore, a more robust study design that includes comparisons between DCE-MRA and other imaging modalities is warranted.

## 7 Conclusion

DCE-MRA is a valuable pre-procedural assessment tool for TAME. This advanced imaging technique enhances the visualization of angiogenesis at the lesion site, offering detailed insights regarding the blood supply, including the identification of supplying vessels, variable branching patterns, and vascular anomalies. By accurately mapping these vascular structures, DCE-MRA assists clinicians in planning and executing TAME procedures with greater precision, ultimately contributing to improved patient outcomes and prevention of complications.

## Data Availability

The original contributions presented in the study are included in the article/supplementary material, further inquiries can be directed to the corresponding author.
